# Internalized HIV Stigma, Depressive Symptoms, and Antiretroviral Therapy Adherence Among Pregnant and Postpartum Women With HIV in Ghana: A Cross‐Sectional Study

**DOI:** 10.1002/hsr2.73426

**Published:** 2026-10-31

**Authors:** Setor K. Sorkpor, Rachel G. A. Thompson, Jerry John Ouner, Brittany L. Lane, Yijiong Yang, Ibrahim Yigit

**Affiliations:** ^1^ College of Nursing Florida State University Tallahassee Florida USA; ^2^ Brain Science and Symptom Management Center Florida State University Tallahassee Florida USA; ^3^ Language Centre, College of Humanities University of Ghana Accra Ghana; ^4^ Africa Interdisciplinary Research Institute Accra Ghana; ^5^ Department of Family Health Care Nursing, School of Nursing University of California, San Francisco San Francisco California USA

**Keywords:** AIDS, Ghana, mental health, perinatal, psychological factors, sub‐Saharan Africa

## Abstract

**Background and Aims:**

Pregnancy and the postpartum period can present substantial psychosocial and HIV‐care challenges for women living with HIV (WLWH), with potential consequences for psychological well‐being, treatment engagement, and maternal and infant health. This study examined whether internalized HIV stigma mediates the relationship between depressive symptoms and antiretroviral therapy (ART) adherence among pregnant and postpartum WLWH in Ghana.

**Methods:**

Structured interviews were administered during antenatal and postnatal care visits in private settings to 176 women receiving ART at 11 public health facilities in the Greater Accra Region. Validated measures were used to assess depressive symptoms, internalized HIV stigma, and self‐reported ART adherence. We tested a simple mediation model with bootstrapped confidence intervals to evaluate the indirect association between depressive symptoms and ART adherence through internalized HIV stigma, adjusting for age.

**Results:**

Approximately one‐third (35.8%) of participants reported below‐average ART adherence. Greater depressive symptom severity was associated with higher internalized HIV stigma, which was associated with poorer adherence. Although depressive symptoms were not directly associated with nonadherence, a statistically significant indirect association was observed through internalized stigma, consistent with an indirect‐only mediation pattern. Age was not significantly associated with either stigma or adherence.

**Conclusion:**

Our findings suggest that internalized HIV stigma may help explain the association between depressive symptoms and reduced ART adherence among pregnant and postpartum WLWH in Ghana. The results support consideration of integrated approaches that address both mental health and stigma within maternal HIV care. Scalable models, including group‐based therapy delivered by lay health workers, may offer opportunities to support ART adherence and well‐being in resource‐limited settings. These findings contribute to our understanding of how psychosocial factors may shape treatment engagement among WLWH and identify potential targets for future intervention research.

**Abbreviations**
ART: Antiretroviral Therapy
IPT‐G: Group Interpersonal Therapy
IPV: Intimate partner violence
WLWH: Women living with HIV

## 1 Introduction

Despite advances in HIV treatment, maintaining antiretroviral therapy (ART) adherence continues to be a challenge for people living with HIV (PLWH) [1, 2], including pregnant and postpartum women living with HIV (WLWH) in sub‐Saharan Africa [3, 4]. Sustained ART adherence is important for achieving viral suppression, preventing mother‐to‐child transmission, and protecting maternal health. However, during the perinatal period, psychosocial factors such as HIV‐related stigma and depression may interfere with adherence [5, 6]. HIV‐related stigma is particularly relevant because it can affect engagement with healthcare and create additional difficulties in maintaining ART use [7]. Within Ghana, structural inequities, constraints in healthcare access, and continuing HIV‐related stigma may further contribute to nonadherence and adverse maternal outcomes among pregnant and postpartum WLWH [8, 9].

For WLWH, pregnancy and the postpartum period can involve the simultaneous demands of HIV treatment, maternal and infant health concerns, and psychosocial stress. These competing demands may affect engagement in HIV care and quality of life [6, 10]. Depression during the perinatal period may also have implications for ART adherence as well as maternal and child health outcomes [6, 11]. In Ghana, maintaining engagement in HIV care presents additional challenges because barriers to retention vary between pregnancy and the postpartum period and include social, economic, stigma‐related, and newborn health factors [8]. These considerations highlight the importance of care approaches that respond to the specific circumstances of pregnant and postpartum women living with HIV.

HIV‐related stigma can operate through several dimensions, including experienced, anticipated, perceived, and internalized stigma, which may influence health behaviors in different ways [5, 12, 13, 14, 15]. Internalized stigma specifically reflects the incorporation of negative societal attitudes about HIV into one's own beliefs and self‐perceptions and may manifest as shame or diminished self‐worth [5, 12]. Higher levels of internalized HIV stigma have been associated with poorer ART adherence among PLWH [16, 17]. Evidence further suggests that internalized stigma may represent an intermediary process through which external forms of stigma, including experienced and anticipated stigma, are associated with adherence behaviors [14, 15, 16, 18]. Despite this evidence, these relationships have received comparatively limited attention among pregnant and postpartum WLWH in sub‐Saharan Africa, where HIV‐related stigma continues to affect WLWH [19, 20].

Depression is another important psychological factor associated with ART adherence, particularly during the perinatal period [6, 21]. Depressive symptoms may interfere with motivation, executive functioning, and self‐efficacy, potentially disrupting medication‐taking routines [22, 23]. Internalized stigma has also been associated with greater depressive symptoms, adding to the psychological burden that may adversely affect adherence [16, 17]. The relationship between depression and internalized stigma may, however, be bidirectional; depressive symptoms may also heighten internalized stigma and contribute to a reinforcing pattern of psychological distress and poorer engagement in care [24]. Against this background, the present study examined whether internalized HIV stigma statistically mediates the association between depressive symptoms and ART nonadherence among pregnant and postpartum WLWH in Ghana. Examining this indirect association may help clarify how depressive symptoms and internalized stigma jointly relate to ART adherence and inform interventions that address both psychological distress and HIV‐related stigma.

## 2 Methods

### 2.1 Study Design and Setting

We conducted a secondary cross‐sectional analysis of data collected between March and May 2022 at 11 public hospitals and health centers in the Greater Accra Region of Ghana. This study is reported in accordance with the Strengthening the Reporting of Observational Studies in Epidemiology (STROBE) guidelines for cross‐sectional studies. The parent dataset and study setting have been described in previous publications [25, 26]. A previous analysis of this dataset examined experienced and anticipated HIV stigma in relation to depressive symptoms through internalized stigma [11]. The present analysis addresses a distinct research question concerning depressive symptoms, internalized stigma, and ART adherence. The participating facilities provide antenatal and postnatal care, including prevention of mother‐to‐child transmission services for WLWH.

### 2.2 Participants and Recruitment

Participants were recruited by convenience sampling from women attending routine antenatal or postnatal care at the participating facilities. Eligibility criteria included age 18 years or older, receipt of ART for at least 2 months, and the ability to provide written informed consent in English or a local language. Women were excluded if clinical records documented intellectual disability or a serious medical or psychiatric condition that could interfere with study participation. Trained research assistants administered the surveys in private areas of the health facilities, using each participant's preferred language.

### 2.3 Data Collection and Ethical Considerations

Study data were collected electronically using tablets and Research Electronic Data Capture (REDCap) [27]. The electronic survey incorporated range and logic checks to minimize data‐entry errors and support data consistency. Research assistants received standardized training before administering the survey across the participating sites. All participants provided written informed consent before study procedures began. The parent study was approved by the University of California San Francisco Institutional Review Board (21‐35733) and the Ghana Health Service Ethics Review Committee (GHS‐ERC: 003/12/21). The current secondary analysis was conducted using de‐identified data from the parent study.

### 2.4 Measures

#### 2.4.1 Depressive Symptoms

Depressive symptoms were measured with the 11‐item Center for Epidemiologic Studies Depression Scale (CES‐D) [28]. Participants indicated how frequently they had experienced each symptom during the previous week using a three‐point response scale: 1 (“Some or a little of the time [1–2 days]”), 2 (“Occasionally or a moderate amount of the time [3–4 days]”), and 3 (“Most or all of the time [5–7 days]”). The measure captures emotional, somatic, and cognitive symptoms. Positively worded items were reverse scored. Item responses were combined, with higher scores indicating more depressive symptoms. Internal consistency in the current sample was acceptable (Cronbach's α = 0.70).

#### 2.4.2 Internalized HIV Stigma

Internalized HIV stigma was assessed with a 16‐item composite measure developed for this study using items drawn from validated instruments, including the Internalized Stigma of AIDS Tool (ISAT) [29]. Participants rated their agreement with each item on a five‐point scale ranging from 1 (“Strongly disagree”) to 5 (“Strongly agree”). Item responses were combined such that increasing scores represented greater internalized HIV stigma. The measure demonstrated good internal consistency in the current sample (Cronbach's α = 0.84).

#### 2.4.3 Medication Adherence

ART adherence was assessed using the Simplified Medication Adherence Questionnaire (SMAQ), a 6‐item validated tool that captures self‐reported behaviors such as forgetfulness, carelessness, and missed doses [30]. Following established scoring criteria, responses were dichotomized as ‘average and above adherence’ versus ‘below average adherence’ if participants endorsed any item indicating nonadherence (e.g., missed doses in the past week, weekend, or past 3 months).

#### 2.4.4 Data Analysis

Descriptive statistics were calculated for the full sample. We tested a simple mediation model specifying depressive symptoms as the independent variable, internalized HIV stigma as the mediator, and ART nonadherence as the dependent variable. A post hoc power analysis was conducted to evaluate the adequacy of the sample size for detecting indirect (mediated) effects with percentile bootstrap [31]. The analysis indicated that detecting a statistically significant indirect effect, assuming medium effect sizes for both the path from the independent variable to the mediator (α = 0.39) and the path from the mediator to the dependent variable (β = 0.39), would require approximately 78 participants to achieve 80% power. Indirect effects were estimated using PROCESS Model 4 in SPSS with 5,000 bootstrap resamples and percentile‐based confidence intervals (CIs). Statistical evidence of an indirect effect was determined by a CI that excluded zero [32]. Models were adjusted for age, and unstandardized coefficients were reported. Additional sociodemographic covariates, including marital status, education, and socioeconomic indicators, were not included due to imbalanced category distributions in this sample, which limited their appropriate inclusion in the mediation models. All statistical tests were two‐sided. Analyses were conducted using IBM SPSS Statistics, version 29.

## 3 Results

Table 1 summarizes the characteristics of the 176 pregnant and postpartum WLWH included in the analysis. Participants had a mean age of 31.91 years (SD = 5.12; range = 18–44). Nearly 69% reported either no monthly income or income below 500 Ghanaian Cedi (GHS), 60.8% (*n* = 107) had attained primary education or less, 55.7% (*n* = 98) were married or separated, and 23.7% (*n* = 40) reported having a current partner living with HIV. Mean scores were 17.41 (SD = 3.56; range = 11–25) for depressive symptoms and 2.61 (SD = 0.91; range = 1–5) for internalized HIV stigma. Regarding ART adherence, 63 participants (35.8%) were classified as nonadherent, while 113 (64.2%) demonstrated average or above‐average adherence.

**Table 1:** Sociodemographic characteristics, Internalized HIV Stigma, depressive symptoms, and SMAQ‐assessed ART Adherence among Study Participants.

Characteristics	Frequency (%)
Age, mean (SD)	31.91 (5.12)
Age groups	
< 30 years	54 (31.0)
30–34 years	64 (36.8)
≥ 35 years	56 (32.2)
Marital status	
Married/separated	98 (55.7)
In a relationship, living/not with a partner	71 (40.3)
Single, never married, no current partner	7 (4.0)
Is current partner HIV‐positive	
Yes	40 (23.7)
No	71 (42.0)
Unknown or unsure	58 (34.3)
Number of children	
None	19 (11.0)
1–2 children	82 (47.4)
3 or more children	72 (41.6)
Religion	
Protestant/Catholic	33 (18.8)
Pentecostal	54 (30.7)
Charismatic	57 (32.4)
Others (Adventist/Muslim)	32 (18.2)
Educational attainment	
Primary education or less	107 (60.8)
Secondary education	42 (23.9)
Higher secondary level, diploma, university degree or higher	27 (15.3)
Educational attainment of partner	
Primary education or less	47 (26.7)
Secondary education	59 (33.5)
Higher secondary level, diploma, university degree or higher	56 (31.8)
Unknown or missing	14 (8.0)
Employment status	
Formal	16 (9.1)
Informal	57 (32.8)
Self‐employed	48 (30.0)
Unemployed	49 (27.8)
Monthly income	
Zero income	39 (22.4)
Below GH₵500	81 (46.0)
GH₵500 or more	56 (31.6)
Place of residence	
Urban	140 (79.5)
Peri‐urban/rural	36 (20.5)
Internalized HIV stigma, mean (SD)	2.61 (0.91)
Depressive symptoms, mean (SD)	17.41 (3.56)
SMAQ‐assessed ART adherence	
Average and above	113 (64.2)
Below average	63 (35.8)

Figure 1 presents the mediation model examining depressive symptoms, internalized HIV stigma, and ART nonadherence. Depressive symptoms were not significantly associated with ART nonadherence when internalized stigma was excluded from the model (B = −0.03, Adjusted Odds Ratio [AOR] = 0.971, SE = 0.04, *p* = 0.513). Consistent with established mediation procedures, a nonsignificant total effect does not preclude evaluation of an indirect effect [32]. The estimated indirect effect through internalized stigma was statistically significant (B = 0.04, SE = 0.02, CI [.0079, 0.0895]). Greater depressive symptoms were associated with higher internalized HIV stigma (B = 0.09, SE = 0.02, *p* < 0.001), and higher internalized stigma was associated with poorer ART adherence (B = 0.46, SE = 0.19, *p* = 0.017). Age was not significantly associated with internalized HIV stigma (B = 0.01, SE = 0.01, *p* = 0.763) or ART nonadherence (B = −0.04, SE = 0.03, *p* = 0.156).

**Figure 1:**
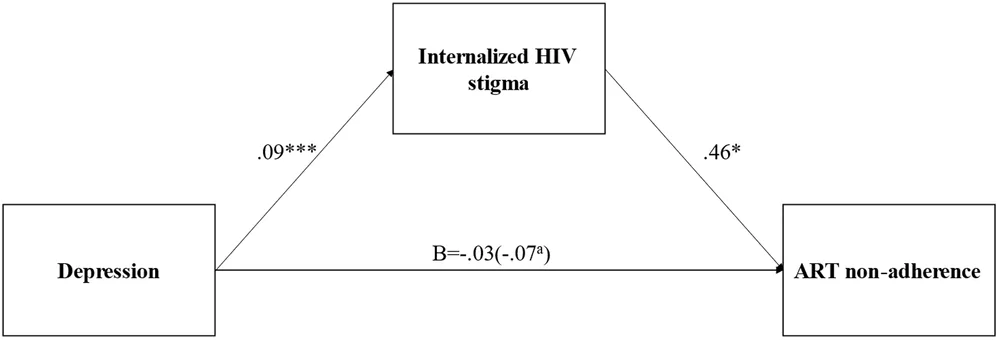
The indirect effect of depressive symptoms on ART nonadherence through internalized HIV stigma. *p < 0.05, ***p < 0.001. ^a^The direct effect when the mediator is included in the model.

## 4 Discussion

The principal finding of this study was a significant indirect association between depressive symptoms and ART nonadherence through internalized HIV stigma among pregnant and postpartum WLWH in Ghana. Although depressive symptoms were not directly associated with ART nonadherence, greater depressive symptoms were associated with higher internalized HIV stigma, and higher internalized stigma was subsequently associated with an increased likelihood of ART nonadherence. This pattern suggests that internalized stigma may help account for the relationship between depressive symptoms and ART adherence during the perinatal period. Pregnant and postpartum WLWH may experience substantial psychosocial stress [33], encounter stigma within community and healthcare settings [19, 34], and face barriers to accessing mental health support [35, 36]. Examining depressive symptoms and stigma together during this period may therefore be important for developing interventions responsive to these intersecting challenges.

While prior studies in sub‐Saharan Africa have identified links among depression, stigma, and ART nonadherence, few have empirically examined the specific indirect relationship tested in this study. Brittain et al reported that internalized stigma was associated with elevated depressive symptoms among pregnant WLWH in South Africa [37], suggesting a strong interconnection between psychological distress and stigma. Similarly, Onono et al found that both depression and stigma were independently associated with reduced ART adherence among postpartum women in Kenya [38]. However, neither study modeled internalized stigma as an intermediary between depression and ART adherence. In contrast to most prior work, Turan et al. proposed and found evidence for a model in which depression mediated the association between stigma and ART adherence [16]. The present analysis examined the variables in the alternative statistical ordering, with depressive symptoms as the predictor, internalized stigma as the mediator, and ART nonadherence as the outcome. The significant indirect association observed in this model provides additional evidence of the close interrelationship among psychological distress, stigma internalization, and treatment adherence during the perinatal period.

This indirect‐only effect is consistent with contemporary mediation frameworks, which emphasize that significant indirect effects can emerge even in the absence of a total or direct association [32]. The findings can also be interpreted within the HIV Stigma Framework [12] and suggest that internalized stigma may help explain the statistical relationship between perinatal depressive symptoms and ART adherence. This association may have implications for intervention development. Approaches directed solely toward depressive symptoms may not address stigma‐related factors that are also associated with treatment behavior. Interventions that incorporate both mental health and stigma‐reduction components, particularly those that are scalable and culturally responsive, therefore warrant consideration for pregnant and postpartum WLWH.

The relationship among depressive symptoms, internalized stigma, and adherence should also be considered within the broader structural and social context of HIV care. Facility‐level stigma, limited mental health resources, and social norms surrounding HIV disclosure may contribute to internalized stigma and difficulties with treatment adherence, as reflected in evidence from Ghana and other sub‐Saharan African settings [8, 9]. These challenges may be particularly acute during pregnancy and the postpartum period, when women often navigate complex ART regimens alongside caregiving responsibilities and fear of social rejection [6, 7, 19]. The Ghana AIDS Commission's 2021–2025 Strategic Plan identifies stigma and mental health challenges as major barriers to maternal retention and engagement in HIV care, calling for the integration of psychosocial support into routine maternal HIV services [39]. However, implementation remains limited, and screening, referral, and psychosocial support systems remain underdeveloped [9, 35].

Intervention strategies for pregnant and postpartum WLWH should account for HIV‐related stigma and disclosure concerns, competing antenatal or postnatal care demands, caregiving responsibilities, and barriers to sustained engagement in HIV care [8, 10]. These challenges differ between pregnancy and the postpartum period, highlighting the value of flexible approaches embedded within existing maternal HIV services [8, 11]. Evidence from other resource‐constrained settings in sub‐Saharan Africa further supports context‐sensitive approaches incorporating peer‐based support and community‐based delivery to address intersecting psychosocial and structural barriers to ART adherence [40, 41]. In settings where specialist mental health resources are limited, task‐shifted models such as Group Interpersonal Therapy (IPT‐G) may offer a feasible and scalable approach. Emerging evidence from sub‐Saharan Africa suggests that IPT‐G can reduce depressive symptoms among young mothers living with HIV and may also help alleviate HIV‐related stigma and psychological distress [42, 43]. Culturally adapted delivery of IPT‐G by lay health workers or peer counselors within maternal HIV care programs warrants further evaluation as an approach to supporting ART adherence and maternal well‐being in resource‐limited settings.

This study contributes to the limited literature examining the mediating role of internalized stigma in the relationship between depressive symptoms and ART nonadherence among perinatal WLWH in Ghana. The use of established measures, a theory‐informed analytic model, and bootstrap estimation of the indirect effect are strengths of the analysis. Several limitations should also be considered. Given that depressive symptoms, internalized stigma, and adherence were assessed cross‐sectionally, the temporal ordering assumed in the mediation model cannot be established, and the indirect association should not be interpreted as evidence of a causal sequence. In addition, internalized stigma, depressive symptoms, and ART adherence were self‐reported and may therefore be affected by reporting or social desirability bias. The study did not comprehensively assess other psychosocial determinants of health that may influence ART adherence. Participants were recruited from public health facilities within a single predominantly urban region of Ghana, which may limit the applicability of the findings to rural populations or to women receiving care in private‐sector settings. Finally, the models included age as a covariate, but imbalanced distributions prevented appropriate adjustment for several other sociodemographic characteristics. Therefore, residual confounding by marital status, education, socioeconomic status, or related factors remains possible.

## 5 Conclusion

In conclusion, a significant indirect association between depressive symptoms and ART nonadherence was observed through internalized HIV stigma among pregnant and postpartum WLWH in Ghana. These findings highlight the value of considering depressive symptoms and internalized HIV stigma together when addressing ART adherence within maternal HIV care. Future research should examine additional psychosocial factors and use longitudinal and intervention designs to determine whether combined approaches targeting mental health and stigma can improve ART adherence and maternal health outcomes in this population.

## Author Contributions

**Setor K. Sorkpor:** writing – original draft, writing – review and editing, formal analysis, methodology, validation. **Rachel G. A. Thompson:** resources, writing – review and editing, writing – original draft, project administration, investigation, validation. **Jerry John Ouner:** conceptualization, funding acquisition, writing – review and editing, supervision, resources, data curation, investigation, validation. **Brittany L. Lane:** writing – original draft, writing – review and editing, validation. **Yijiong Yang:** formal analysis, writing – original draft, writing – review and editing, methodology, validation. **Ibrahim Yigit:** writing – original draft, methodology, writing – review and editing, formal analysis, validation.

## Ethics Statement

The study was conducted in accordance with the Declaration of Helsinki and received approval from the University of California San Francisco Institutional Review Board (21‐35733) and the Ghana Health Service Ethics Review Committee (GHS‐ERC: 003/12/21). All participants provided written informed consent during the original data collection. The present study involved secondary analysis of de‐identified parent‐study data.

## Conflicts of Interest

The authors declare no conflicts of interest.

## Transparency Statement

The lead author, Setor K. Sorkpor, affirms that this manuscript is an honest, accurate, and transparent account of the study being reported; that no important aspects of the study have been omitted; and that any discrepancies from the study as planned (and, if relevant, registered) have been explained.

## Data Availability

The data that support the findings of this study are available from the corresponding author upon reasonable request. All authors have read and approved the final version of the manuscript. Ibrahim Yigit had full access to all of the data in this study and takes complete responsibility for the integrity of the data and the accuracy of the data analysis.

## References

[1] M. J. Mugavero , K. R. Amico , T. Horn , and M. A. Thompson , “The State of Engagement in HIV Care in the United States: From Cascade to Continuum to Control,” Clinical Infectious Diseases 57, no. 8 (2013): 1164–1171, 10.1093/cid/cit420. 23797289

[2] N. Nwangwu‐Ike , E. L. Frazier , N. Crepaz , Y. Tie , and M. Y. Sutton , “Racial and Ethnic Differences in Viral Suppression Among HIV‐Positive Women in Care,” JAIDS Journal of Acquired Immune Deficiency Syndromes 79, no. 2 (2018): e56–e68. 10.1097/QAI.0000000000001779 30212433

[3] R. M. Burke , H. M. Rickman , C. Pinto , P. Ehrenkranz , A. Choko , and N. Ford , “Reasons for Disengagement from Antiretroviral Care in the Era of “Treat All” in Low‐ or Middle‐Income Countries: A Systematic Review,” Journal of the International AIDS Society 27, no. 3 (2024): e26230, 10.1002/jia2.26230. PMC10945039 38494657

[4] S. Padmakar , V. Chaudhary , S. Kumari , D. Dhir , and B. Pal , “Prevalence of Suicidal Ideation and Associated Factors Among Perinatal Women Living With HIV: A Systematic Review and Meta‐Analysis,” AIDS Care, no. 3 (2025): 362–371, 10.1080/09540121.2025.2453664. 39864900

[5] B. Turan , K. L. Stringer , M. Onono , et al., “Linkage to HIV Care, Postpartum Depression, and HIV‐Related Stigma in Newly Diagnosed Pregnant Women Living With HIV in Kenya: A Longitudinal Observational Study,” BMC Pregnancy and Childbirth 14 (2014): 400, 10.1186/s12884-014-0400-4. PMC4261547 25467187

[6] A. Kaida , L. T. Matthews , S. Ashaba , et al., “Depression During Pregnancy and the Postpartum Among HIV‐Infected Women on Antiretroviral Therapy in Uganda,” JAIDS Journal of Acquired Immune Deficiency Syndromes 67, no. Suppl 4 (2014): S179–S187, 10.1097/qai.0000000000000370. PMC4251908 25436816

[7] W. Z. Worku , T. Azale , T. A. Ayele , and D. K. Mekonnen , “Women's Voices: The Lived Experience of HIV‐Positive Women in the Postpartum Period at the University of Gondar Comprehensive Specialized Referral Hospital, Ethiopia: A Phenomenological Study,” BMC Women's Health 23, no. 1 (2023): 325, 10.1186/s12905-023-02470-5. PMC10283323 37344854

[8] K. S. Sakyi , M. Y. Lartey , C. E. Kennedy , et al., “Barriers to Maternal Retention in HIV Care in Ghana: Key Differences During Pregnancy and the Postpartum Period,” BMC Pregnancy and Childbirth 20, no. 1 (2020): 398, 10.1186/s12884-020-03067-8. PMC7366890 32677910

[9] J. J. Nutor , A. O. Gyamerah , H. O. Duah , et al., “The Association of HIV‐Related Stigma and Psychosocial Factors and HIV Treatment Outcomes Among People Living With HIV in the Volta Region of Ghana: A Mixed‐Methods Study,” PLOS Global Public Health 4, no. 2 (2024): e0002994, 10.1371/journal.pgph.0002994. PMC10903797 38422060

[10] S. Ashaba , A. Kaida , J. N. Coleman , et al., “Psychosocial Challenges Facing Women Living With HIV during the Perinatal Period in Rural Uganda,” PLoS One 12, no. 5 (2017): e0176256, 10.1371/journal.pone.0176256. PMC5411062 28459866

[11] S. K. Sorkpor , I. Yigit , R. G. A. Thompson , Y. Yang , and J. J. Ouner , “Experienced and Anticipated HIV‐Related Stigma and Depression Among Pregnant and Postpartum Women Living With HIV in the Greater Accra Region of Ghana: The Role of Internalized HIV Stigma,” BMC Public Health 25, no. 1 (2025): 3939, 10.1186/s12889-025-24950-z. PMC12619392 41239309

[12] V. A. Earnshaw and S. R. Chaudoir , “From Conceptualizing to Measuring HIV Stigma: A Review of HIV Stigma Mechanism Measures,” AIDS and Behavior 13, no. 6 (2009): 1160–1177, 10.1007/s10461-009-9593-3. PMC4511707 19636699

[13] B. Turan , A. M. Hatcher , S. D. Weiser , M. O. Johnson , W. S. Rice , and J. M. Turan , “Framing Mechanisms Linking HIV‐Related Stigma, Adherence to Treatment, and Health Outcomes,” American Journal of Public Health 107, no. 6 (2017): 863–869, 10.2105/ajph.2017.303744. PMC5425866 28426316

[14] I. Yigit , B. Turan , G. Kurt , et al., “Longitudinal Associations of Experienced and Perceived Community Stigma With Antiretroviral Therapy Adherence and Viral Suppression in New‐to‐Care People With HIV: Mediating Roles of Internalized Stigma and Depression Symptoms,” JAIDS Journal of Acquired Immune Deficiency Syndromes 95, no. 3 (2024): 231–237, 10.1097/QAI.0000000000003360. PMC10922854 38180894

[15] I. Yigit , R. Paulino‐Ramírez , J. Waters , D. M. Long , J. M. Turan , and H. Budhwani , “A Moderated Mediation Analysis of HIV and Intersectional Stigmas and Antiretroviral Adherence in People Living With HIV in the Dominican Republic,” AIDS and Behavior 28, no. 10 (2024): 3258–3269, 10.1007/s10461-024-04425-9. PMC11524671 38916689

[16] B. Turan , W. S. Rice , K. B. Crockett , et al., “Longitudinal Association between Internalized HIV Stigma and Antiretroviral Therapy Adherence for Women Living With HIV: The Mediating Role of Depression,” AIDS 33, no. 3 (2019): 571–576, 10.1097/qad.0000000000002071. PMC6362840 30702521

[17] I. Yigit , Y. Bayramoglu , S. D. Weiser , et al., “Changes in Internalized Stigma and HIV Health Outcomes in Individuals New to HIV Care: The Mediating Roles of Depression and Treatment Self‐Efficacy,” AIDS Patient Care and STDs 34, no. 11 (2020): 491–497, 10.1089/apc.2020.0114. PMC7698993 33147084

[18] I. Yigit , T. E. Wilson , T. N. Taylor , et al., “Association of Experienced Stigma in Healthcare Settings With Health Outcomes Among Black Women Living With HIV: Mediating Roles of Internalized Stigma, Anticipated Stigma, and Trust in HIV Care,” Social Science & Medicine (1982) 366 (2025): 117699, 10.1016/j.socscimed.2025.117699. PMC11956749 39823813

[19] D. S. Boakye , M. Setordzi , G. Dzansi , and S. Adjorlolo , “Mental Health Burden Among Females Living With HIV and AIDS in Sub‐Saharan Africa: A Systematic Review,” PLOS Global Public Health 4, no. 2 (2024): e0002767, 10.1371/journal.pgph.0002767. PMC10833589 38300927

[20] K. Peltzer , V. J. Rodriguez , T. K. Lee , and D. Jones , “Prevalence of Prenatal and Postpartum Depression and Associated Factors Among HIV‐Infected Women in Public Primary Care in Rural South Africa: A Longitudinal Study,” AIDS Care 30, no. 11 (2018): 1372–1379, 10.1080/09540121.2018.1455960. PMC6150830 29575943

[21] N. L. Mbatha , K. E. Mokwena , and S. Madiba , “Clinical and Obstetric Risk Factors for Postnatal Depression in HIV Positive Women: A Cross Sectional Study in Health Facilities in Rural KwaZulu‐Natal,” International Journal of Environmental Research and Public Health 17, no. 22 (2020): 8425. 10.3390/ijerph17228425 PMC7697934 33202528

[22] A. C. Tsai , D. R. Bangsberg , S. M. Kegeles , et al., “Internalized Stigma, Social Distance, and Disclosure of HIV Seropositivity in Rural Uganda,” Annals of Behavioral Medicine 46, no. 3 (2013): 285–294, 10.1007/s12160-013-9514-6. PMC3795853 23690283

[23] J. S. Gonzalez , A. W. Batchelder , C. Psaros , and S. A. Safren , “Depression and HIV/AIDS Treatment Nonadherence: A Review and Meta‐Analysis,” JAIDS Journal of Acquired Immune Deficiency Syndromes 58, no. 2 (2011): 181–187, 10.1097/QAI.0b013e31822d490a. PMC3858003 21857529

[24] B. Turan , W. Smith , M. H. Cohen , et al., “Mechanisms for the Negative Effects of Internalized HIV‐Related Stigma on Antiretroviral Therapy Adherence in Women: The Mediating Roles of Social Isolation and Depression,” JAIDS Journal of Acquired Immune Deficiency Syndromes 72, no. 2 (2016): 198–205, 10.1097/qai.0000000000000948. PMC4868649 26885803

[25] E. K. Ameyaw , J. J. Nutor , J. Okiring , et al., “The Role of Social Support in Antiretroviral Therapy Uptake and Retention Among Pregnant and Postpartum Women Living With HIV in the Greater Accra Region of Ghana,” BMC Public Health 24, no. 1 (2024): 540, 10.1186/s12889-024-18004-z. PMC10882784 38383341

[26] J. J. Nutor , J. Okiring , I. Yeboah , et al., “Association Between Water Insecurity and Antiretroviral Therapy Adherence Among Pregnant and Postpartum Women in Greater Accra Region of Ghana,” PLOS Global Public Health 4, no. 1 (2024): e0002747, 10.1371/journal.pgph.0002747. PMC10773961 38190403

[27] P. A. Harris , R. Taylor , R. Thielke , J. Payne , N. Gonzalez , and J. G. Conde , “Research Electronic Data Capture (REDCap)—A Metadata‐Driven Methodology and Workflow Process for Providing Translational Research Informatics Support,” Journal of Biomedical Informatics 42, no. 2 (2009): 377–381, 10.1016/j.jbi.2008.08.010. PMC2700030 18929686

[28] L. S. Radloff , “The CES‐D Scale: A Self‐Report Depression Scale for Research in the General Population,” Applied Psychological Measurement 1, no. 3 (1977): 385–401, 10.1177/014662167700100306.

[29] K. D. Phillips , L. Moneyham , and A. Tavakoli , “Development of an Instrument to Measure Internalized Stigma in Those With HIV/AIDS,” Issues in Mental Health Nursing 32, no. 6 (2011): 359–366, 10.3109/01612840.2011.575533. 21692574

[30] H. Knobel , J. Alonso , J. L. Casado , et al., “Validation of a Simplified Medication Adherence Questionnaire in a Large Cohort of HIV‐Infected Patients: The GEEMA Study,” AIDS 16, no. 4 (2002): 605–613, 10.1097/00002030-200203080-00012. 11873004

[31] M. S. Fritz and D. P. Mackinnon , “Required Sample Size to Detect the Mediated Effect,” Psychological Science 18, no. 3 (2007): 233–239, 10.1111/j.1467-9280.2007.01882.x. PMC2843527 17444920

[32] A. F. Hayes , “Beyond Baron and Kenny: Statistical Mediation Analysis in the New Millennium,” Communication Monographs 76, no. 4 (2009): 408–420, 10.1080/03637750903310360.

[33] E. L. Tuthill , A. E. Maltby , B. C. Odhiambo , et al., “I Found Out I Was Pregnant, and I Started Feeling Stressed”: A Longitudinal Qualitative Perspective of Mental Health Experiences Among Perinatal Women Living With HIV,” AIDS and Behavior 25, no. 12 (2021): 4154–4168, 10.1007/s10461-021-03283-z. PMC8126180 33997940

[34] K. S. Sakyi , M. Y. Lartey , C. E. Kennedy , et al., “Stigma Toward Small Babies and Their Mothers in Ghana: A Study of the Experiences of Postpartum Women Living With HIV,” PLoS One 15, no. 10 (2020): e0239310, 10.1371/journal.pone.0239310. PMC7567350 33064737

[35] L. Gyimah , I. A. Agyepong , D. Owiredu , et al., “Tools for Screening Maternal Mental Health Conditions in Primary Care Settings in Sub‐Saharan Africa: Systematic Review,” Frontiers in Public Health 12 (2024): 1321689, 10.3389/fpubh.2024.1321689. PMC11466175 39391163

[36] E. M. Stringer , S. Meltzer‐Brody , M. Kasaro , et al., “Depression, Pregnancy, and HIV: The Case to Strengthen Mental Health Services for Pregnant and Post‐Partum Women in Sub‐Saharan Africa,” Lancet Psychiatry 1, no. 2 (2014): 159–162, 10.1016/S2215-0366(14)70273-1. 26360580

[37] K. Brittain , C. A. Mellins , T. Phillips , et al., “Social Support, Stigma and Antenatal Depression Among HIV‐Infected Pregnant Women in South Africa,” AIDS and Behavior 21, no. 1 (2017): 274–282, 10.1007/s10461-016-1389-7. PMC6116836 27052843

[38] M. Onono , T. Odwar , L. Abuogi , et al., “Effects of Depression, Stigma and Intimate Partner Violence on Postpartum Women's Adherence and Engagement in HIV Care in Kenya,” AIDS and Behavior 24, no. 6 (2020): 1807–1815, 10.1007/s10461-019-02750-y. PMC7228848 31813076

[39] Ghana AIDS Commission . *National HIV and AIDS Strategic Plan 2021–2025*. 2022, https://www.ghanaids.gov.gh/media/docs/NATIONAL‐HIV‐AIDS‐STRATEGIC‐PLAN‐2021‐2025.pdf.

[40] S. K. Sorkpor , I. Yigit , R. G. A. Thompson , B. L. Lane , G. Anguyo , and J. J. Ouner , “Stigma, Food Insecurity, and Limited Social Support as Psychosocial Correlates of Antiretroviral Therapy Adherence Among Pregnant Women Living With HIV: Cross‐Sectional Study,” JMIR Public Health and Surveillance 12 (2026): e84914, 10.2196/84914. PMC13120754 42044489

[41] J. Kabami , L. B. Balzer , S. Kabageni , et al., “Peer‐Mother Counseling Improves HIV Treatment Adherence and Status Disclosure Over Time Among Pregnant and Postpartum Women in Rural Uganda,” AJE Advances: Research in Epidemiology 1, no. 1 (2025): 2, 10.1093/ajeadv/uuaf002.

[42] O. Yator , G. John‐Stewart , L. Khasakhala , and M. Kumar , “Preliminary Effectiveness of Group Interpersonal Psychotherapy for Young Kenyan Mothers With HIV and Depression: A Pilot Trial,” American Journal of Psychotherapy 75, no. 2 (2022): 89–96, 10.1176/appi.psychotherapy.20200050. 34915725

[43] O. Yator , L. Khasakhala , G. J. Stewart , and M. Kumar , “Acceptability and Impact of Group Interpersonal Therapy (IPT‐G) on Kenyan Adolescent Mothers Living With Human Immunodeficiency Virus (HIV): A Qualitative Analysis,” BMC Women's Health 22, no. 1 (2022): 240, 10.1186/s12905-022-01807-w. PMC9206094 35717156

